# Supplementary material for: The interactome of KRAB zinc finger proteins reveals the evolutionary history of their functional diversification
Source: EMBO J. 2019 Aug 12;38(18):e101220. doi: 10.15252/embj.2018101220 (PMC6745500; doi:10.15252/embj.2018101220)
Supplement: Supplementary file 1 — Appendix [file EMBJ-38-e101220-s001.pdf]

Appendix table of contents

Figure S1. KZFPs clustered interactome
Figure S2. Interactions between DUF3669-containing KZFPs in published data sets
Figure S3. Unusual IF patterns
Figure S4. TE subfamilies enriched for the binding of KZFPs associated with more than three unique interactors
Figure S5. Bait KZFPs endogenous expression in 293T cells and human tissues

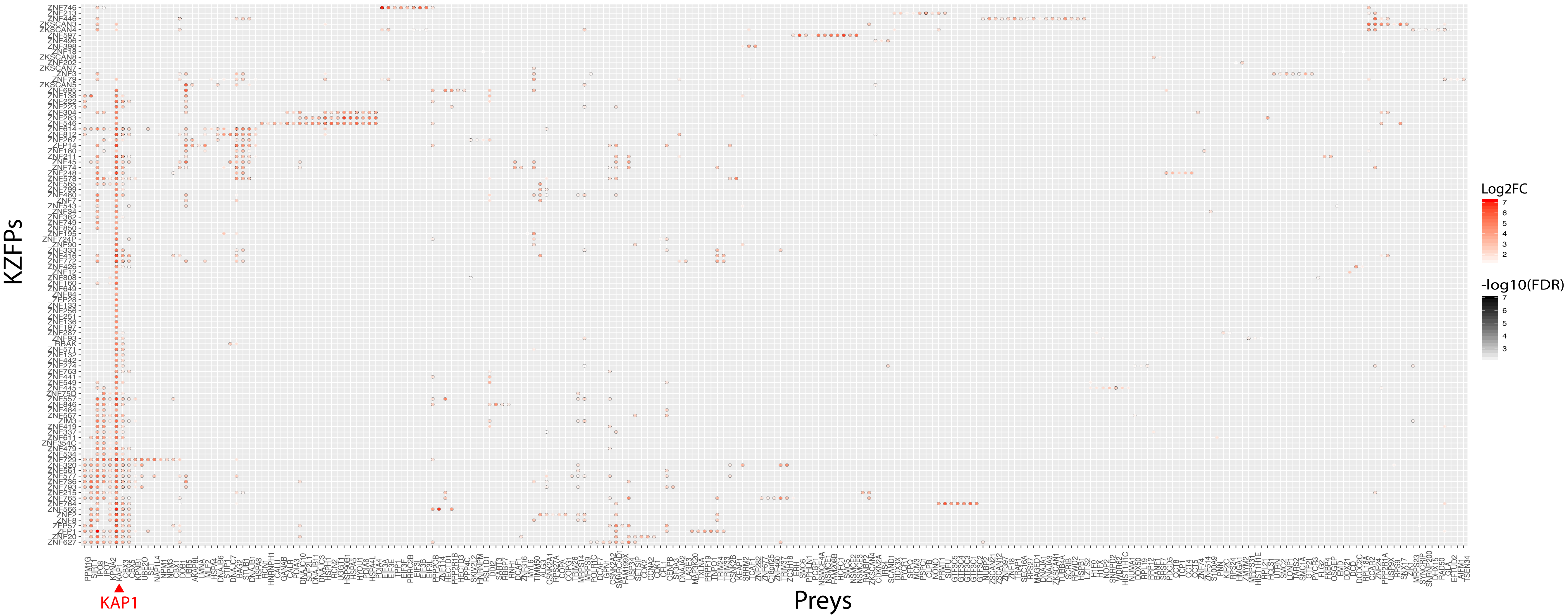

Appendix Figure S1. KZFPs clustered interactome

The KZFP interactome clustered by Pearson correlation. Log2FC = Average of the logged fold changes of spectral counts over controls, FDR = false discovery rate.

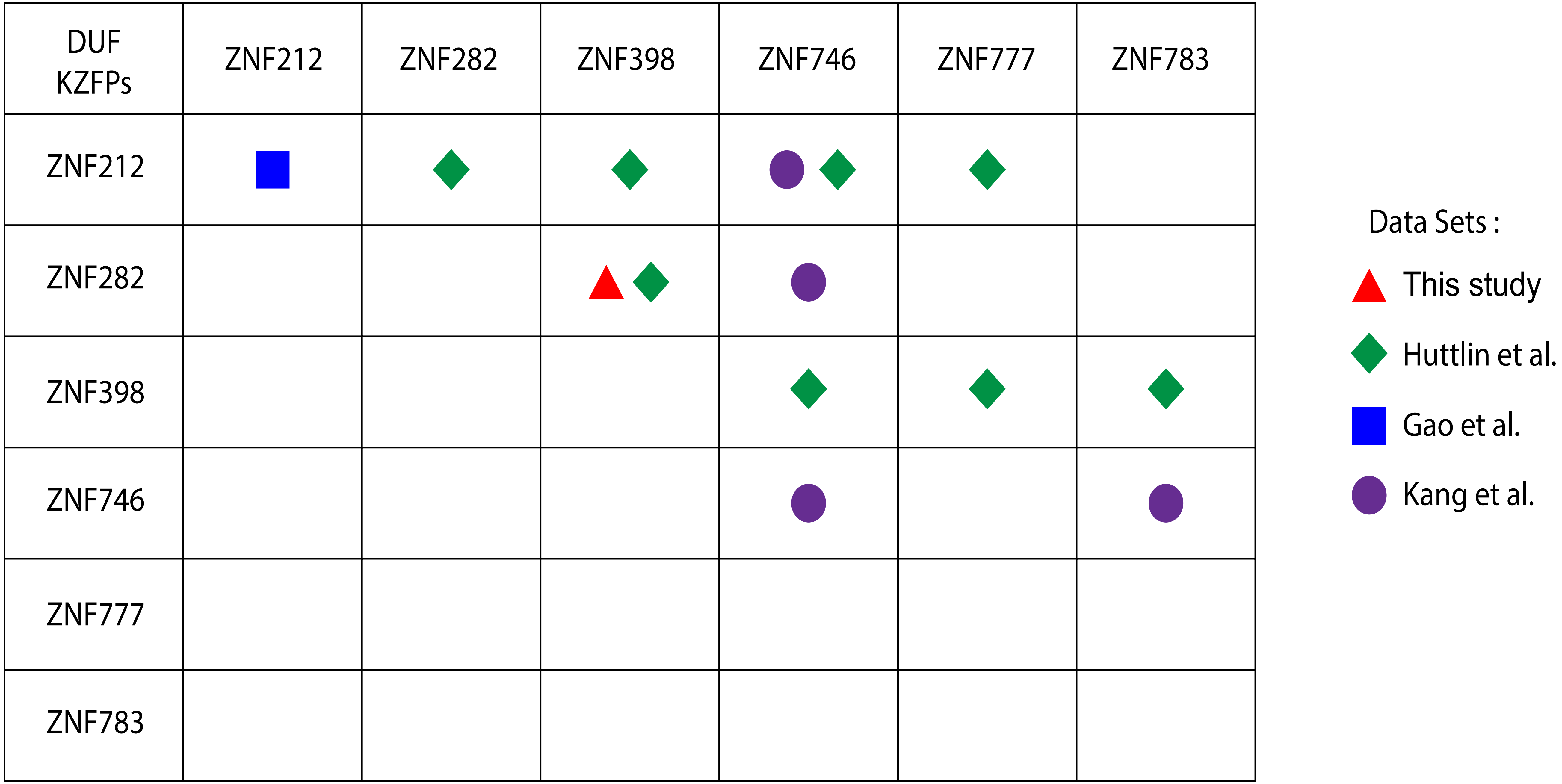

Appendix Figure S2. Interactions between DUF3669-containing KZFPs in published data sets
Grid representing detected interactions between DUF3669-KZFPs in published data sets.

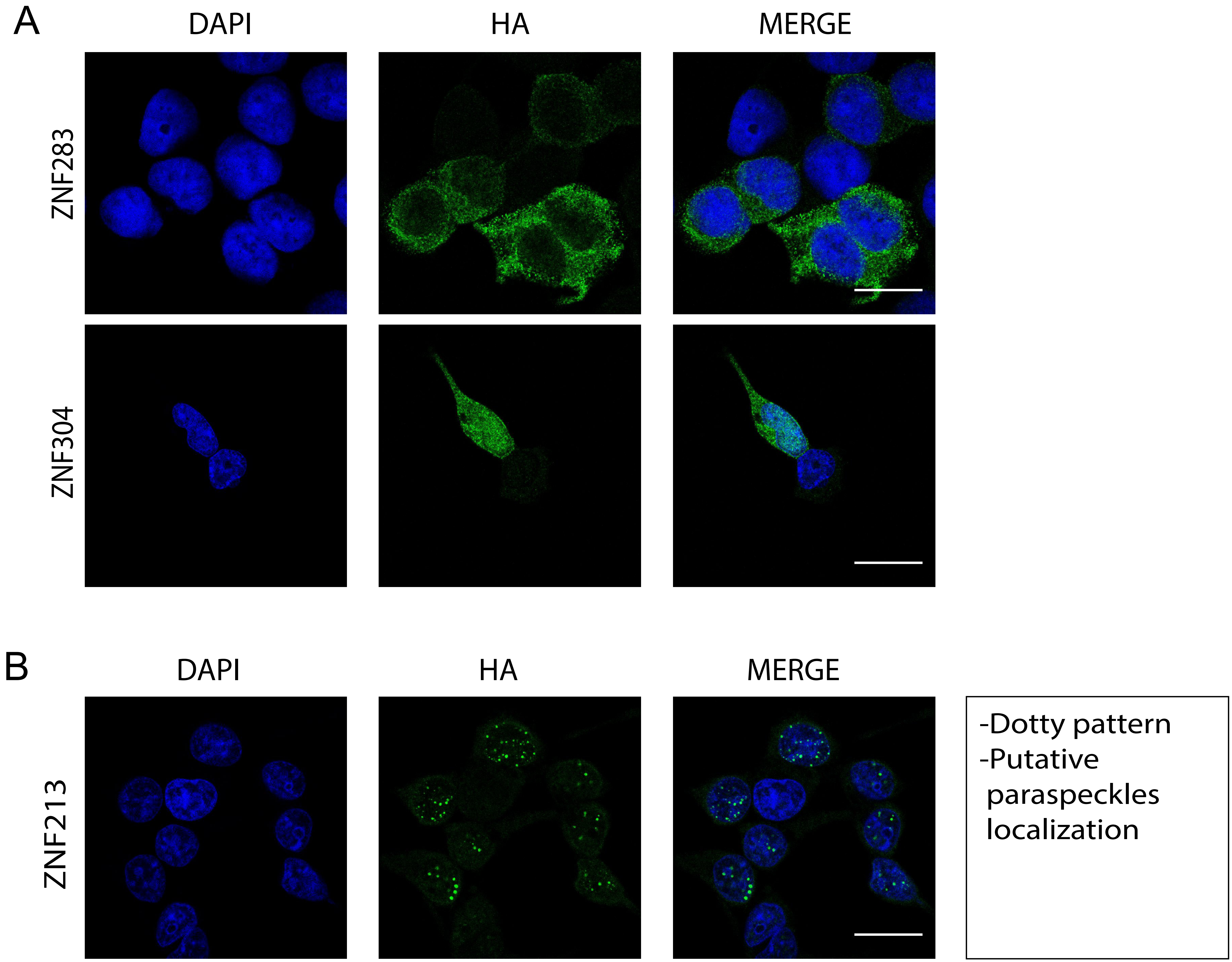

Appendix Figure S3. Unusual IF patterns
A & B. IF by confocal microscopy displaying the different nuclear patterns of mostly cytoplasmic ZNF283 and ZNF304 (A) and putative paraspeckles-associated ZNF213 (B). Staining was performed with anti-HA (Alexa-488, green) and DNA was stained with Dapi (blue). The scale bar represents 20 μm.

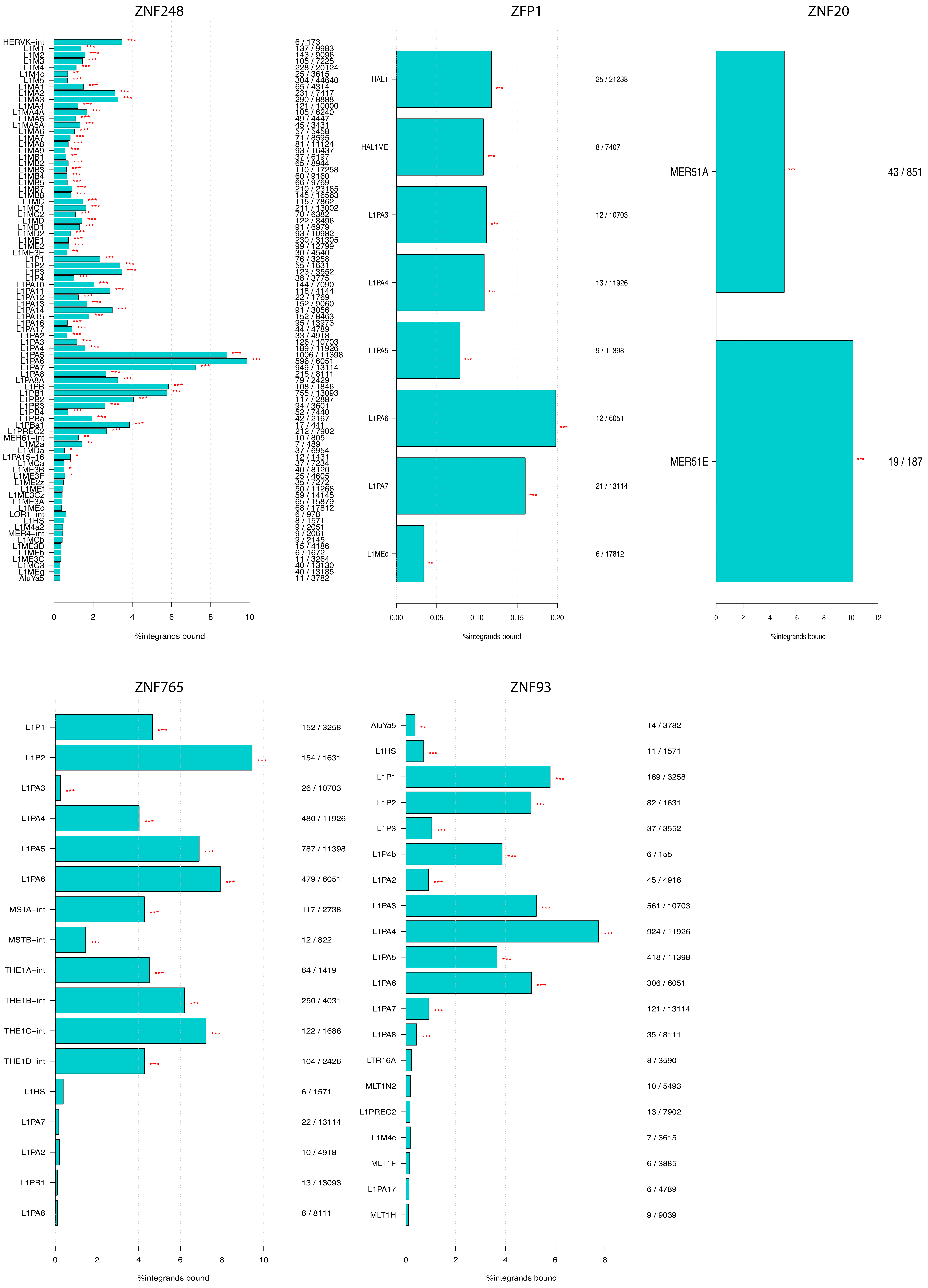

Appendix Figure S4. TE subfamilies enriched for the binding of KZFPs associated with more than three unique interactors

TE subfamilies enriched for the binding of KZFPs associated with more than three unique interactors. P values for the TE families enrichment were computed using an hypergeometric test and were corrected for multiple testing using the Benjamini & Yekutieli method.

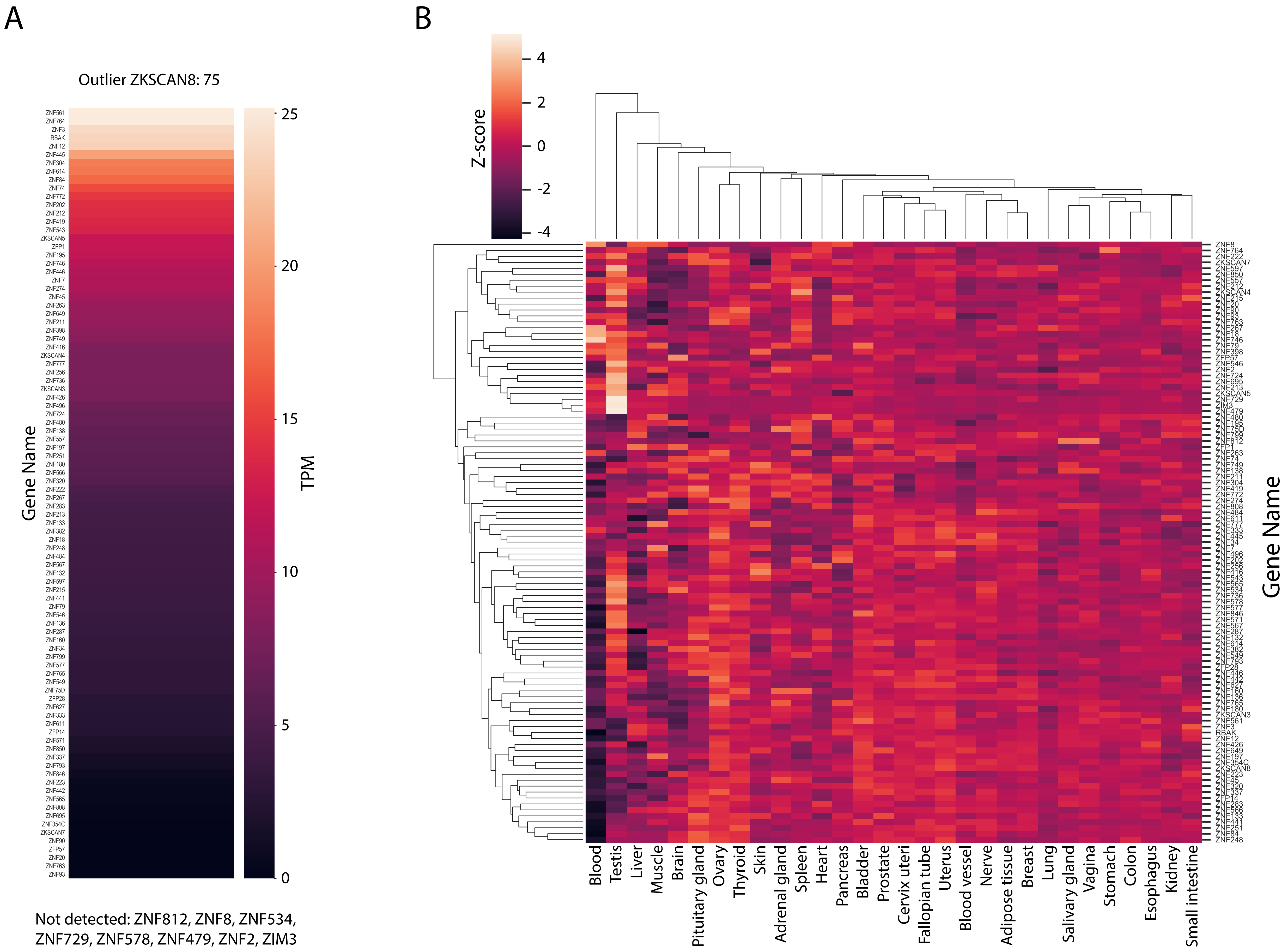

Appendix Figure S5. Bait KZFPs endogenous expression in 293T cells and human tissues

A. Expression heatmap of bait-KZFPs derived from an in-house RNA-sequencing experiment performed on 293T cells, TPM = transcripts per kilobase million. ZKSCAN8 high value was excluded from this map for clarity. Bait-KZFPs for which transcripts were not detected are indicated at the bottom. B. Expression (Z-score per row) clustermap of bait-KZFPs in a variety of human tissues (The GTEx consortium, 2013).
