# Supplementary material for: The interactome of KRAB zinc finger proteins reveals the evolutionary history of their functional diversification
Source: EMBO J. 2019 Aug 12;38(18):e101220. doi: 10.15252/embj.2018101220 (PMC6745500; doi:10.15252/embj.2018101220)
Supplement: Supplementary file 2 — Expanded View Figures PDF [file EMBJ-38-e101220-s002.pdf]

Expanded View Figures

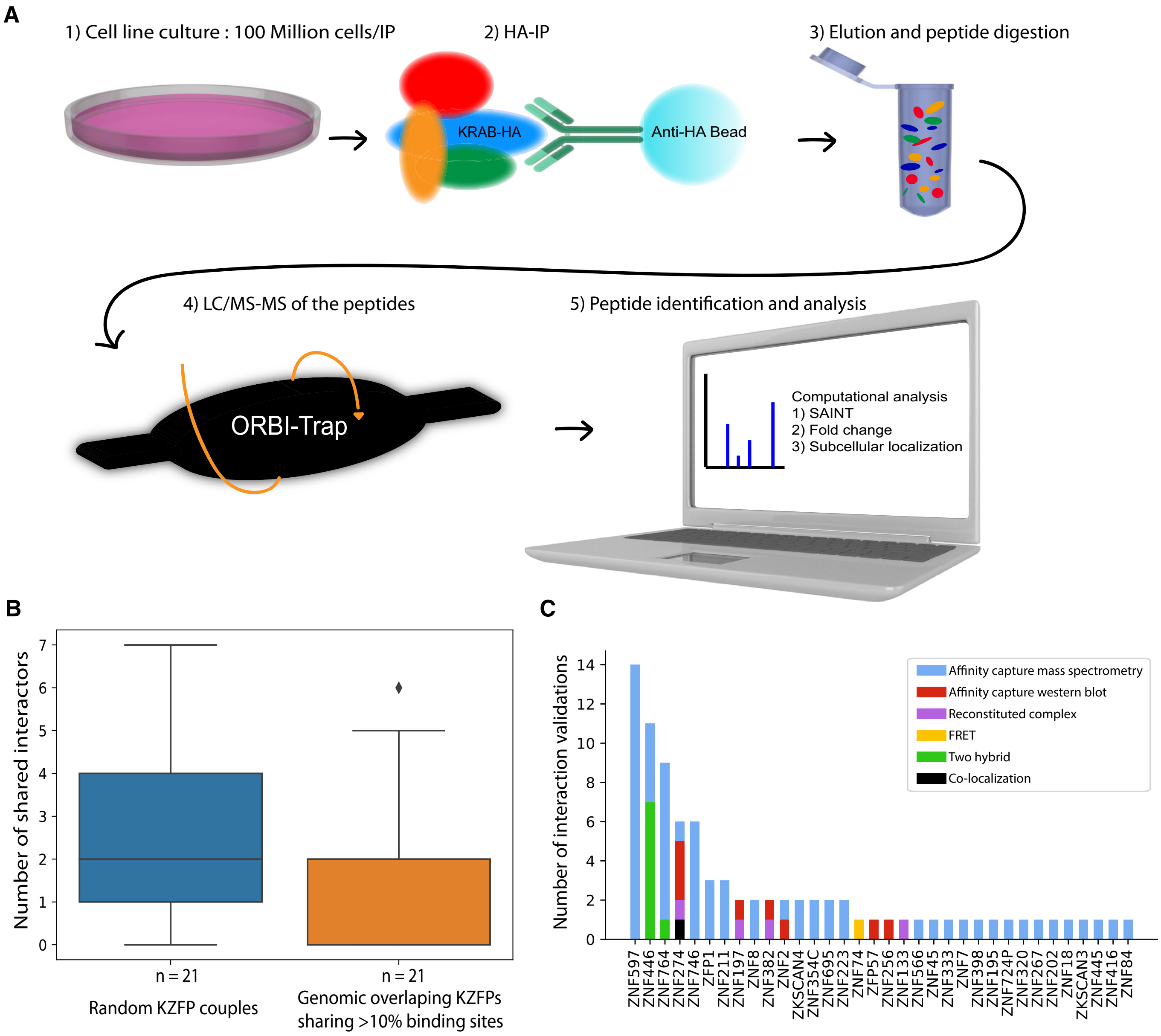

Figure EV1. General AP-MS protocol and quality controls.

A Graphical representation of AP-MS protocol major steps and filters used for the computational analysis.

B Boxplot presenting the number of shared interactors for KZFP couples sharing more than 10% of their binding sites in a reciprocal fashion compared to the number of shared interactors for the same amount of random KZFP couples. Boxplots are shown as median, and 25th (Q1) and 75th (Q3) percentiles. The upper whisker extends to the last data point less than Q3 + 1.5*IQR, where IQR = Q3–Q1. Similarly, the lower whisker extends to the first data point greater than Q1 – 1.5*IQR.

C Number of interactions observed in previous studies (Table EV2) associated with their corresponding KZFP. The method used for the interaction detection is indicated by a specific color.

© 2019 The Authors

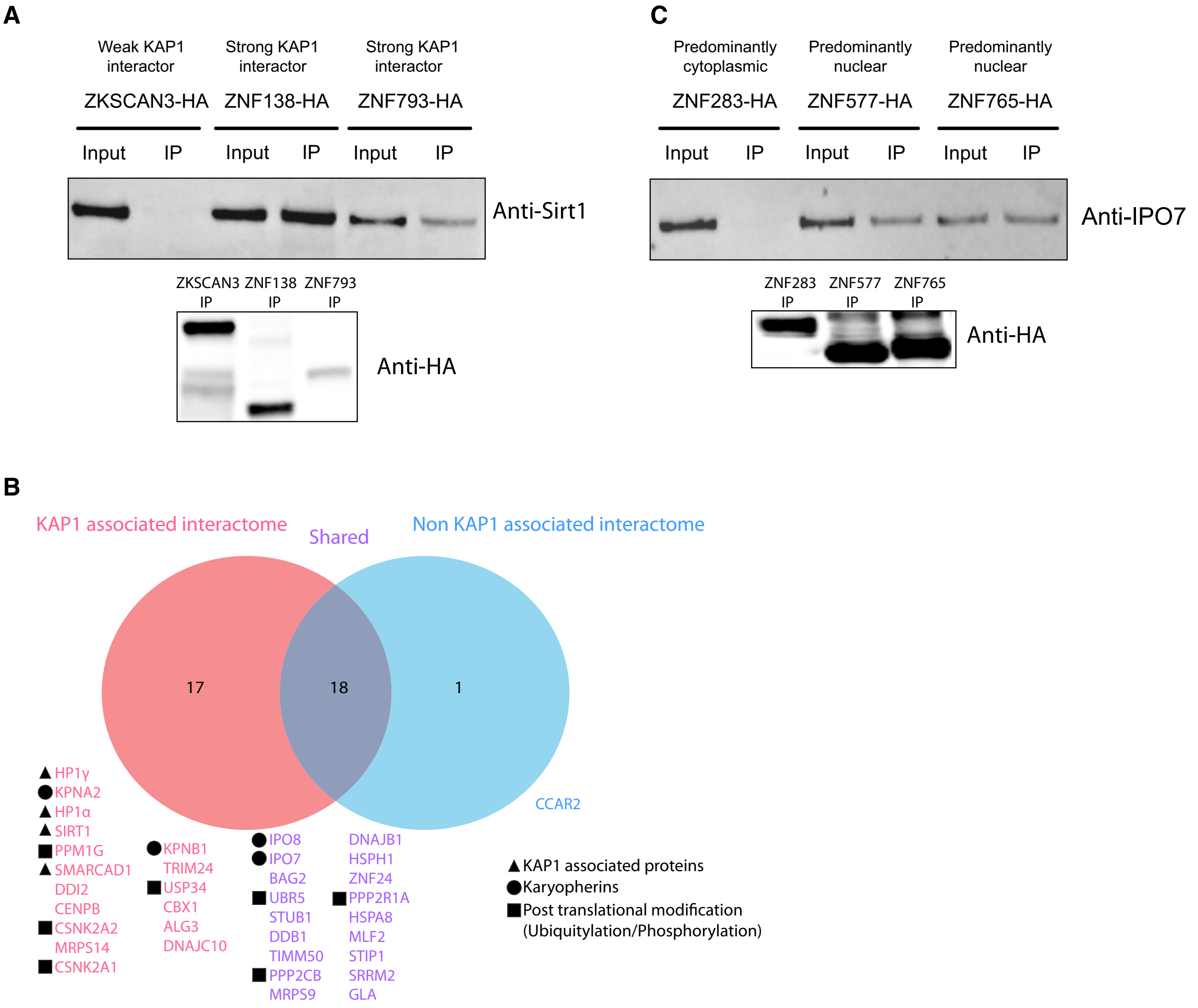

Figure EV2. KZFP common interactors.

A SIRT1 interaction validation. HA-tagged KZFPs transduced cell lines were used for immunoprecipitations. HA immunoprecipitation of weak KAP1 interactor ZKSCAN3 and candidates ZNF138 and ZNF793, followed by the detection of endogenous SIRT1 in the immunoprecipitates through Western blot using an anti-SIRT1 antibody. Input = cellular lysate, IP = immunoprecipitate. Western blot using an anti-HA antibody on the IPs at the bottom.

B Venn diagram representing all the common preys that were detected in 5 and more KZFP interactomes. In pink are shown the common preys that only appear in interactomes alongside KAP1, in blue the prey that only appears in interactomes devoid of KAP1, and in purple the preys that are in both type of interactomes.

C IPO7 interaction validation. HA-tagged KZFPs transduced cell lines were used for immunoprecipitations. HA immunoprecipitation of predominantly cytoplasmic ZNF283 and candidates ZNF577 and ZNF765, followed by the detection of endogenous IPO7 through Western blot using an anti-IPO7 antibody. Input = cellular lysate, IP = immunoprecipitate. Western blot using an anti-HA antibody on the IPs at the bottom.

Source data are available online for this figure.

© 2019 The Authors

Figure EV3. Impact of the composition, age, and structure of the KRAB domain on KAP1 recruitment.

A Swarm plot representing the observed range of KAP1FC values as well as the determination of a strong KAP1 interaction threshold of 3.

B Double representation of KAP1 interaction for selected strong and weak KAP1-interacting KZFPs. Top: Experimentally determined KAP1FC values are represented by a histogram. Bottom: KAP1 interaction determined by immunoprecipitations. HA-tagged KZFPs transduced cell lines were used for immunoprecipitations. Input = cellular lysate, IP = immunoprecipitate. HA immunoprecipitation of KZFPs followed by endogenous KAP1 detection through Western blot using an anti-KAP1 antibody. Western blot using an anti-HA antibody on the IPs at the bottom.

C Violin plot representing the KAP1FC values for KZFPs in function of the presence of a B-box in their KRAB domain.

D Simple phylogenetic tree representing the time of divergences of coelacanth and different tetrapod clades from the *Homo sapiens* lineage.

E Evolutionary age of all sKZFPs and vKZFPs present in the UniProt database and our previous study (Imbeault *et al*, 2017). Mann–Whitney two-sided rank test.

Data information: Boxplots are shown as median, and 25th (Q1) and 75th (Q3) percentiles. The upper whisker extends to the last data point less than Q3 + 1.5*IQR, where IQR = Q3–Q1. Similarly, the lower whisker extends to the first data point greater than Q1 – 1.5*IQR.
Source data are available online for this figure.

© 2019 The Authors

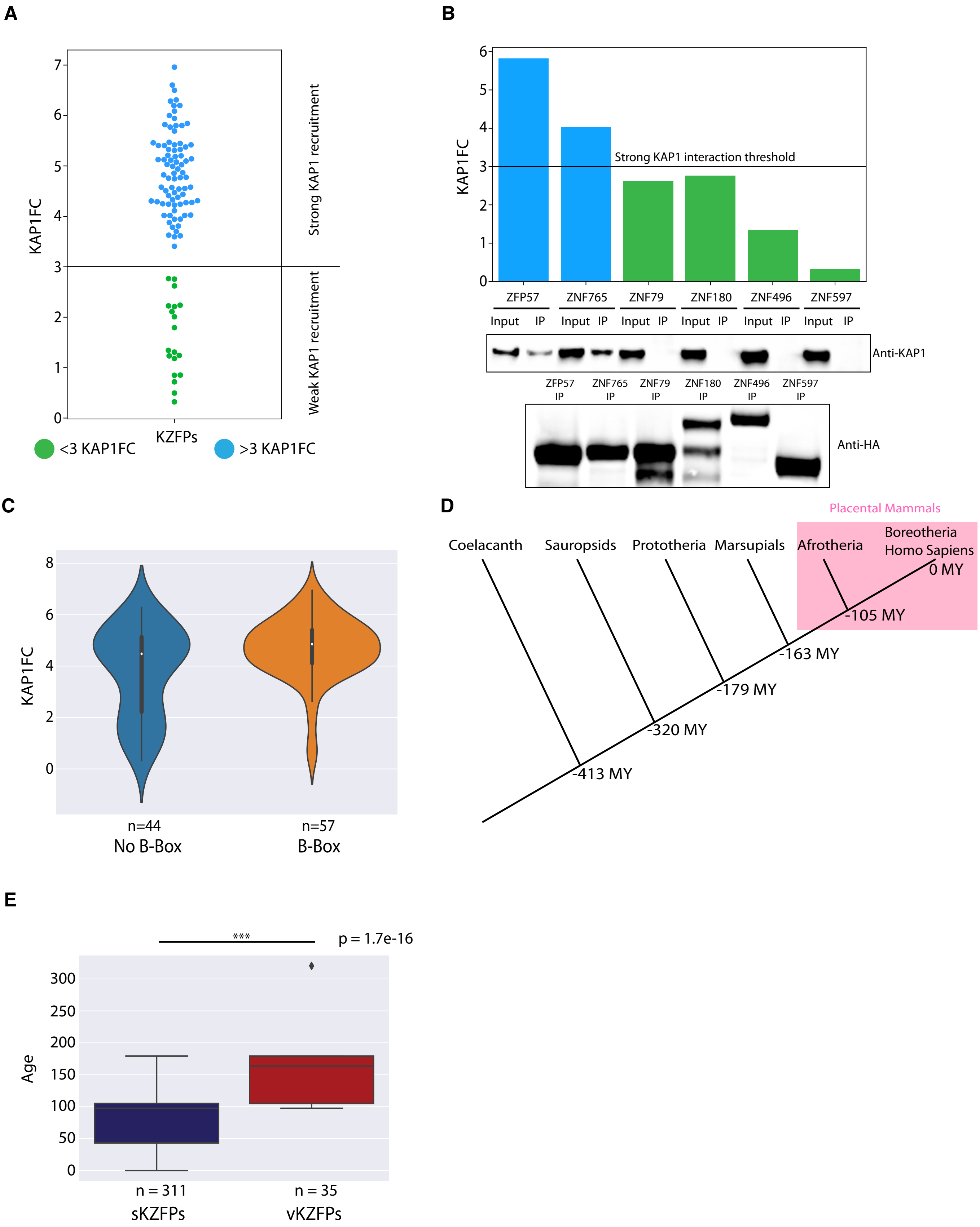

Figure EV3.

 © 2019 The Authors

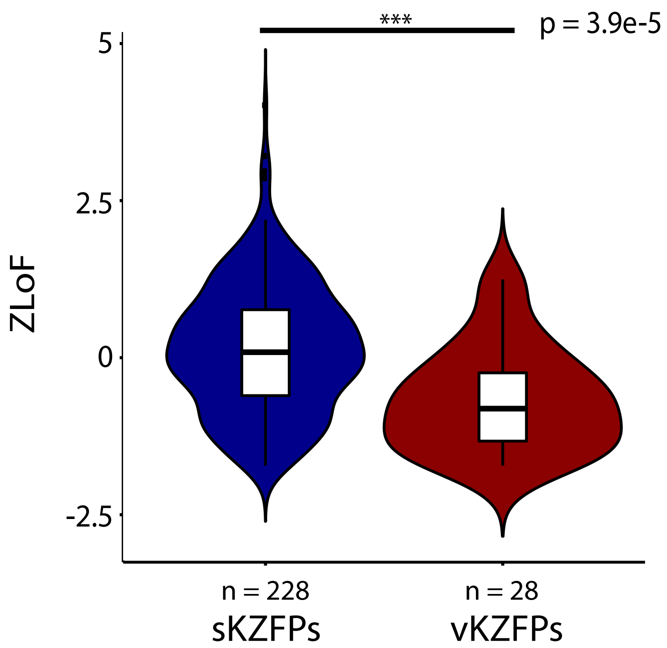

Figure EV4. vKZFPs loss-of-function mutations in the human population.

Z-score for the number of loss-of-function mutations found in the canonical transcripts (ZLoF) of vKZFP or sKZFP based on human exome and whole genome data obtained from the gnomAD database. Unpaired Wilcoxon test. Data information: Boxplots are shown as median, and 25th (Q1) and 75th (Q3) percentiles. The upper whisker extends to the last data point less than Q3 + 1.5*IQR, where IQR = Q3–Q1. Similarly, the lower whisker extends to the first data point greater than Q1 – 1.5*IQR.

© 2019 The Authors

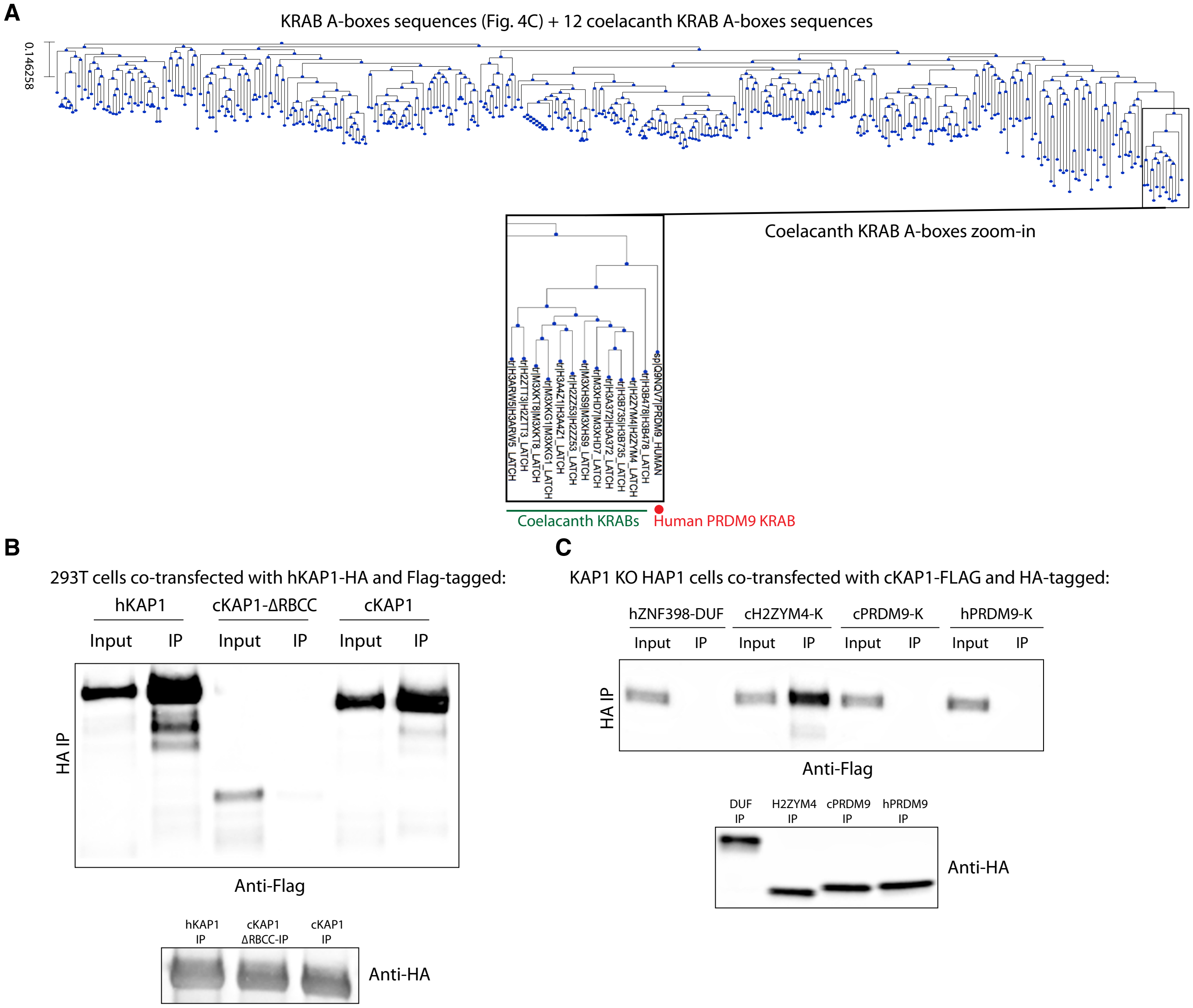

Figure EV5. Human and coelacanth KRAB domains.

- A Phylogenetic tree built based on amino acid sequence alignment of human KZFP KRAB A-boxes and UniProt-determined coelacanth KZFP KRAB A-boxes domains, bottom: zoom-in on the cluster containing all of the coelacanth KRAB domains, coelacanth KRAB domains are indicated in green while hPRDM9 KRAB is indicated in red.
- B Immunoprecipitation of HA-tagged hKAP1 domain in order to check interaction with cKAP1. Co-transfection of HA-tagged hKAP1 and Flag-tagged hKAP1, negative control ΔRBCC cKAP1 (the RBCC domain mediates KAP1 oligomerization), and cKAP1 in 293T cells followed by HA immunoprecipitation. The presence of different KAP1 constructs was revealed by Western blot using an anti-Flag antibody. Input = cellular lysate, IP = immunoprecipitate. Western blot using an anti-HA antibody on the IPs at the bottom.
- C Immunoprecipitation of HA-tagged human and coelacanth PRDM9 KRAB domains in order to check interaction with cKAP1. Co-transfection of Flag-tagged cKAP1 and HA-tagged ZNF398 DUF3669 domain, H2ZYM4 cKRAB domain, and PRDM9 hKRAB and cKRAB domains in KAP1 KO HAP1 cells followed by HA immunoprecipitation. cKAP1 presence was revealed by Western blot using an anti-Flag antibody. Input = cellular lysate, IP = immunoprecipitate. Western blot using an anti-HA antibody on the IPs at the bottom.

Source data are available online for this figure.

 © 2019 The Authors
