# Supplementary figures and images for: The interactome of KRAB zinc finger proteins reveals the evolutionary history of their functional diversification
Source: EMBO J. 2019 Aug 12;38(18):e101220. doi: 10.15252/embj.2018101220 (PMC6745500; doi:10.15252/embj.2018101220)

A

Sirt1 on IPs

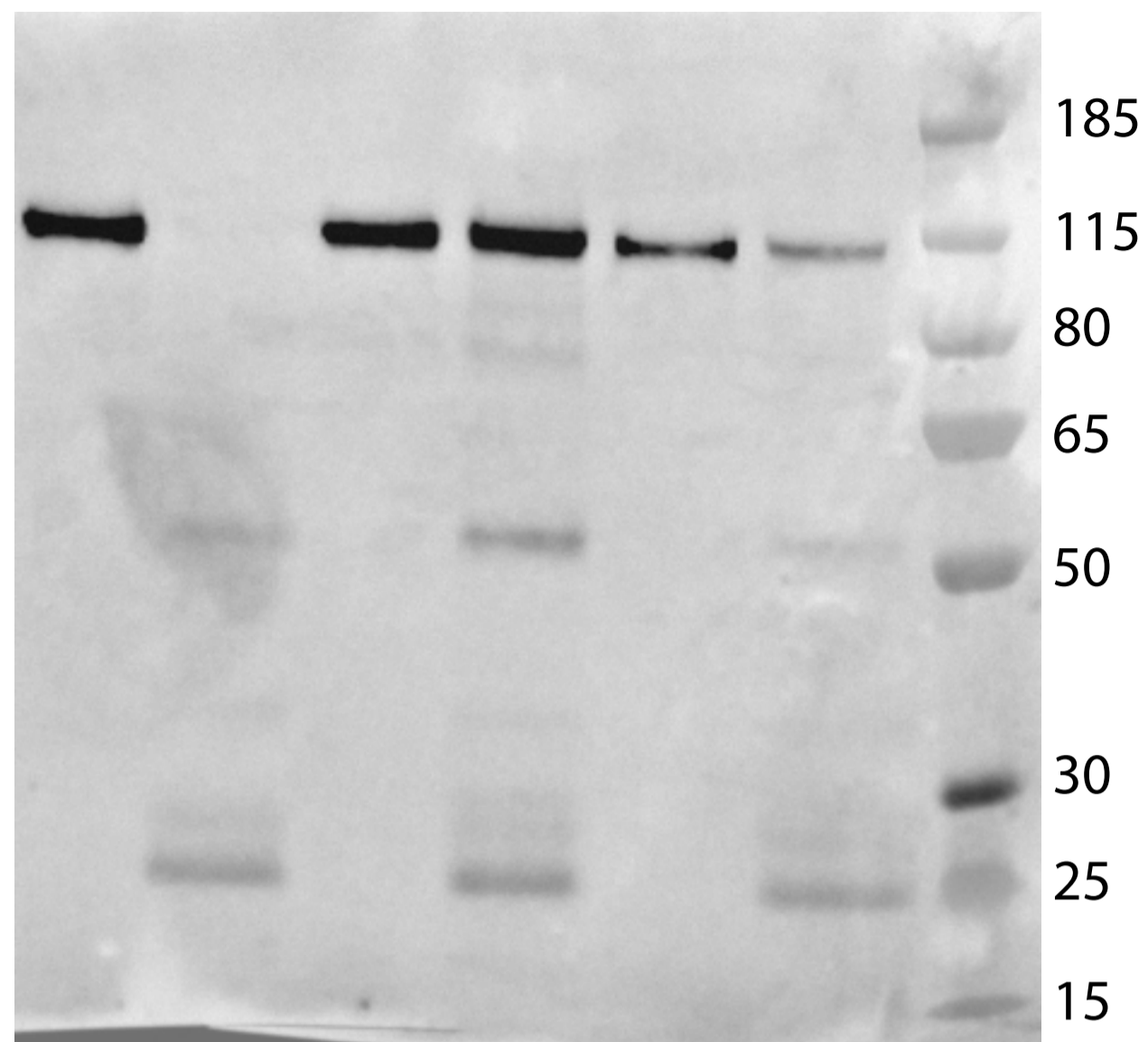

HA on IPs

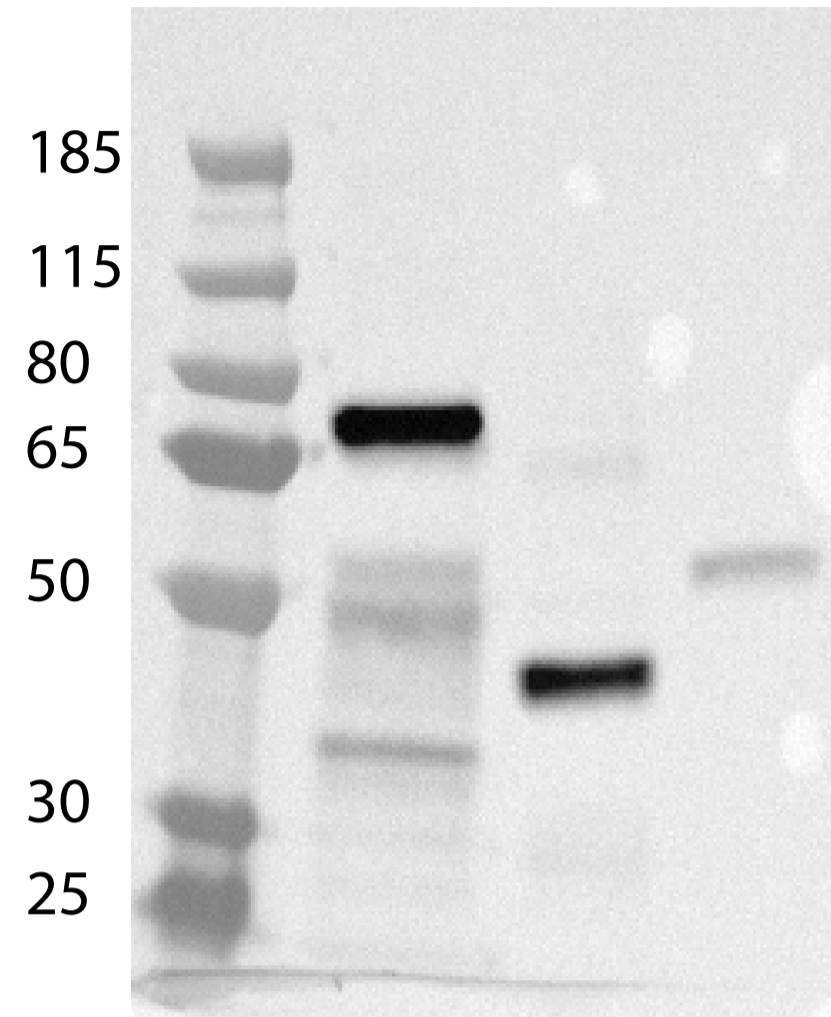

C

IPO7 on IPs

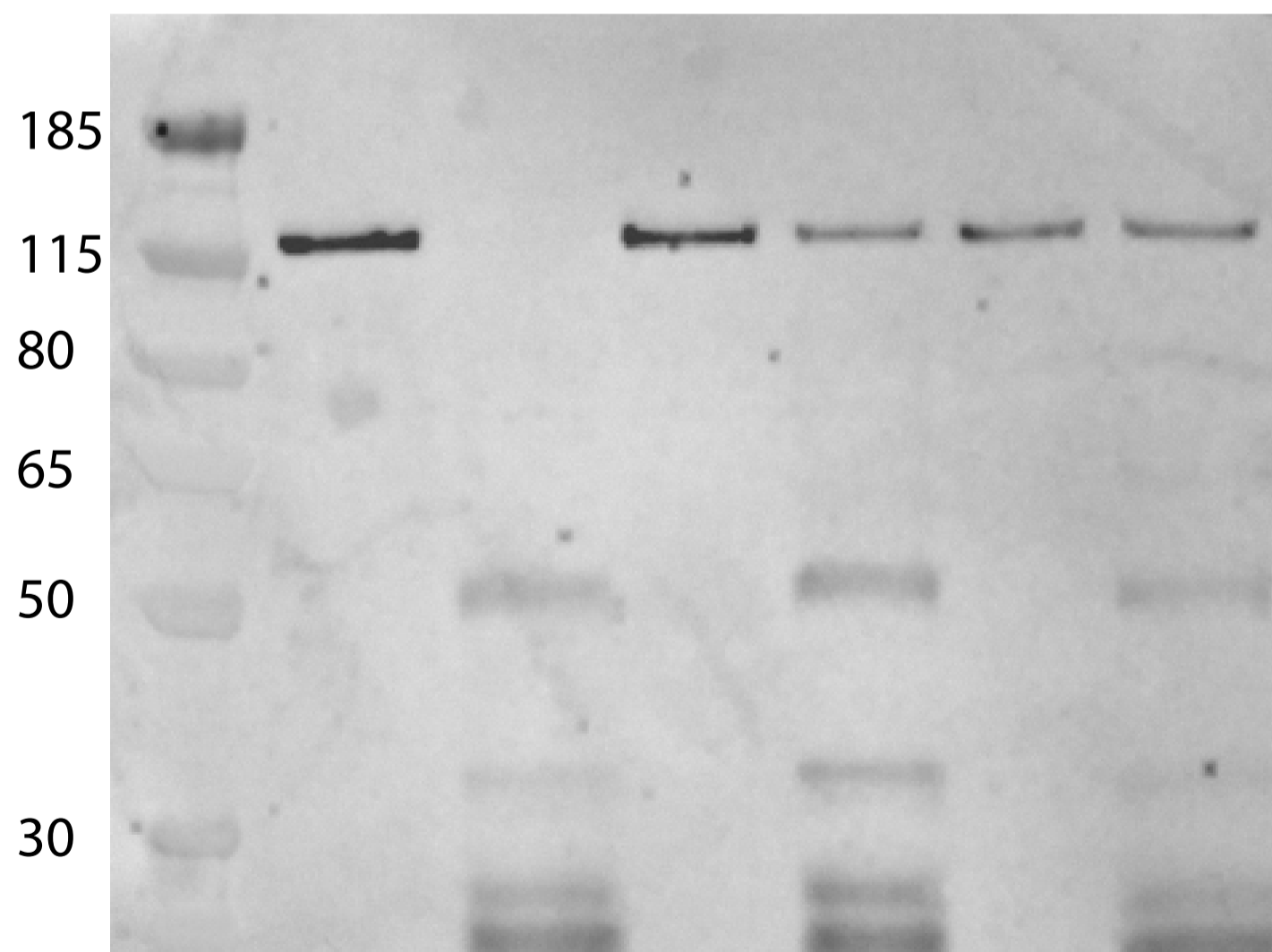

HA on IPs

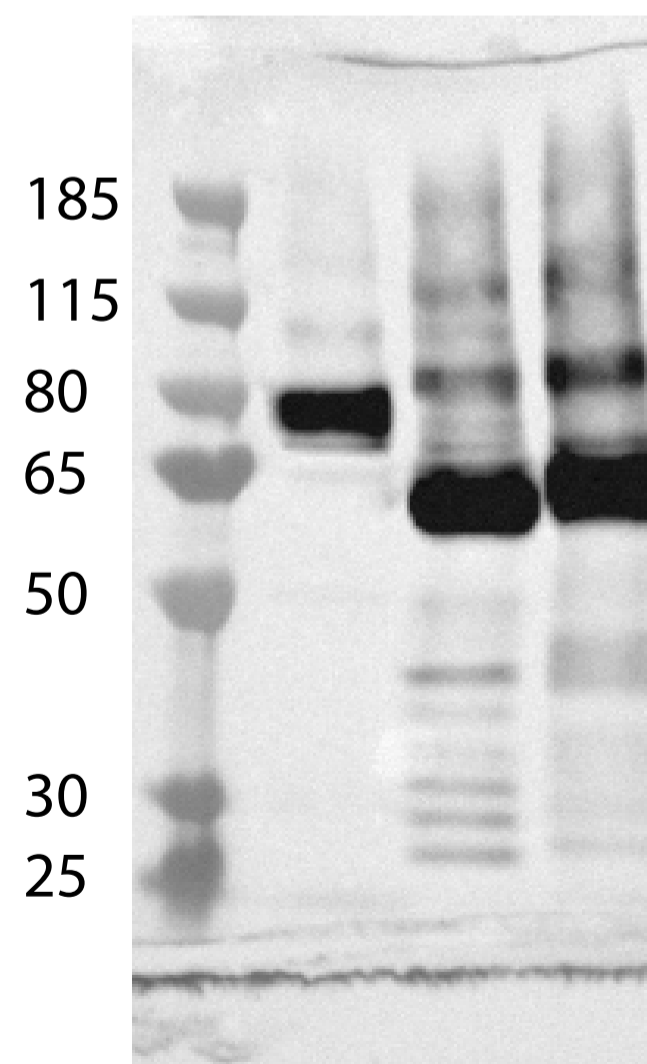

Supplement: Supplementary file 7 — Source Data for Expanded View [file EMBJ-38-e101220-s009.zip › Source_Data_Fig_EV2.pdf]

B

### KAP1 on IPs

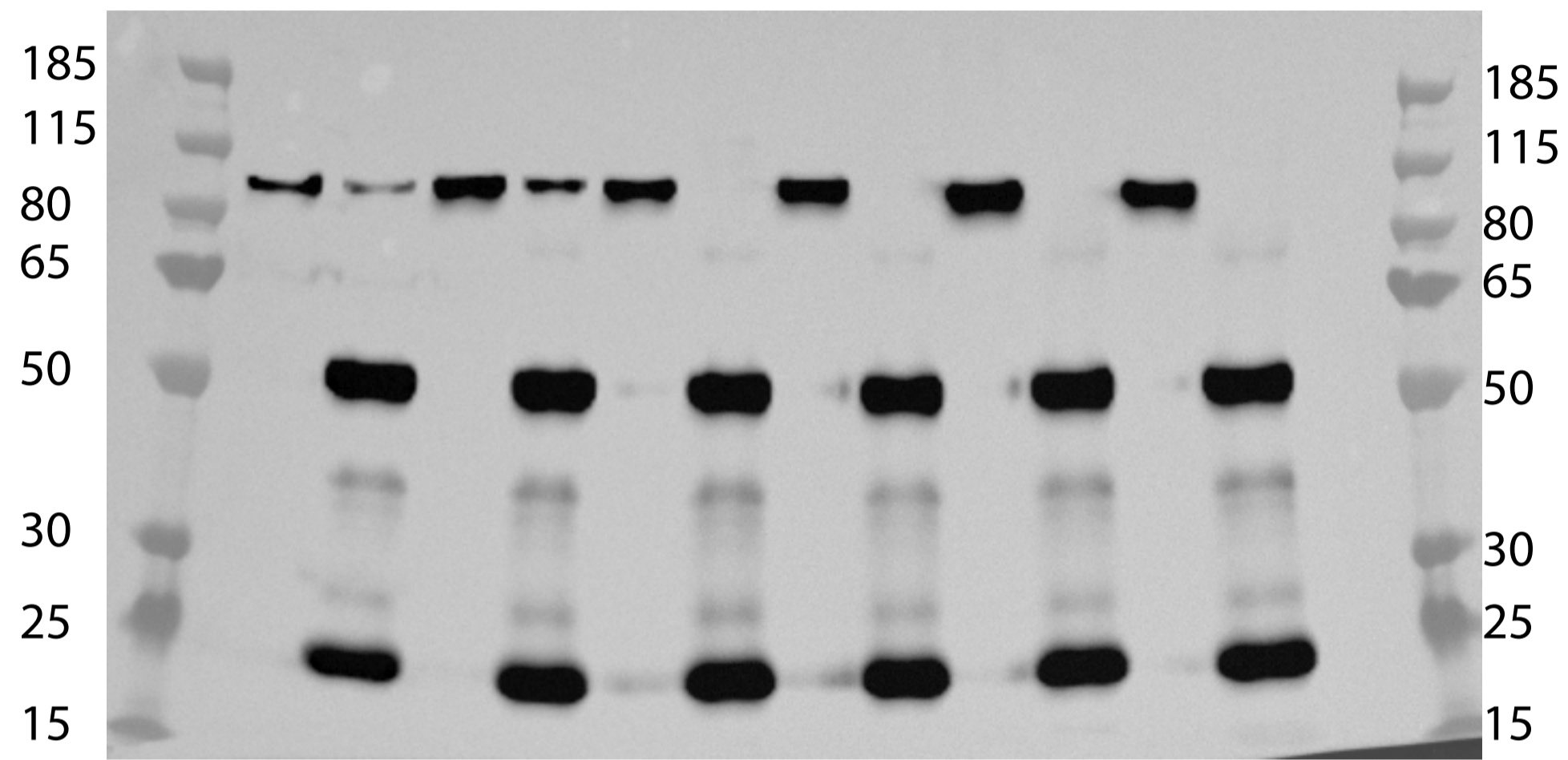

### HA on IPs

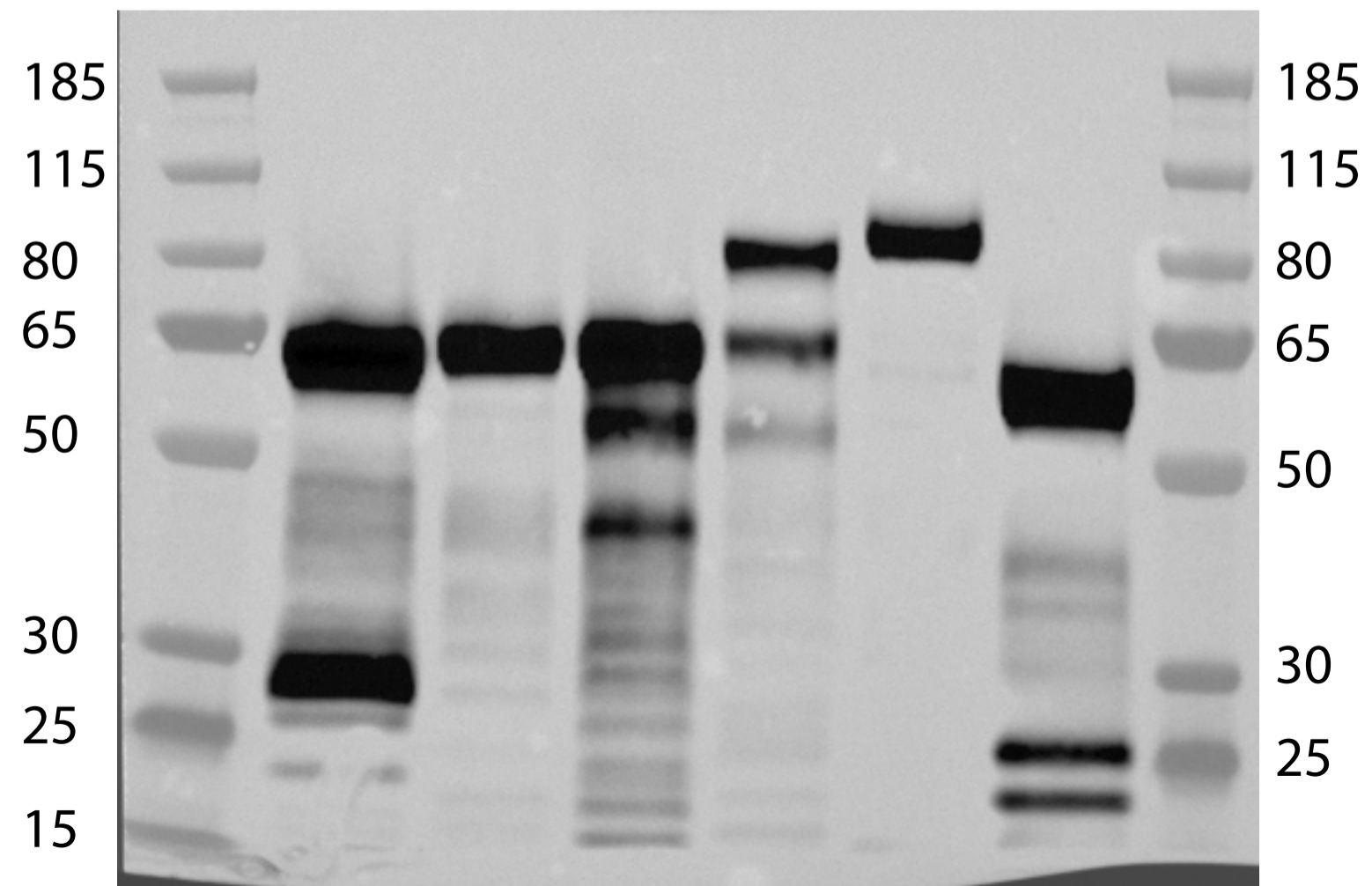

Supplement: Supplementary file 7 — Source Data for Expanded View [file EMBJ-38-e101220-s009.zip › Source_Data_Fig_EV3.pdf]

B

Anti-FLAG

Anti-HA on IPs

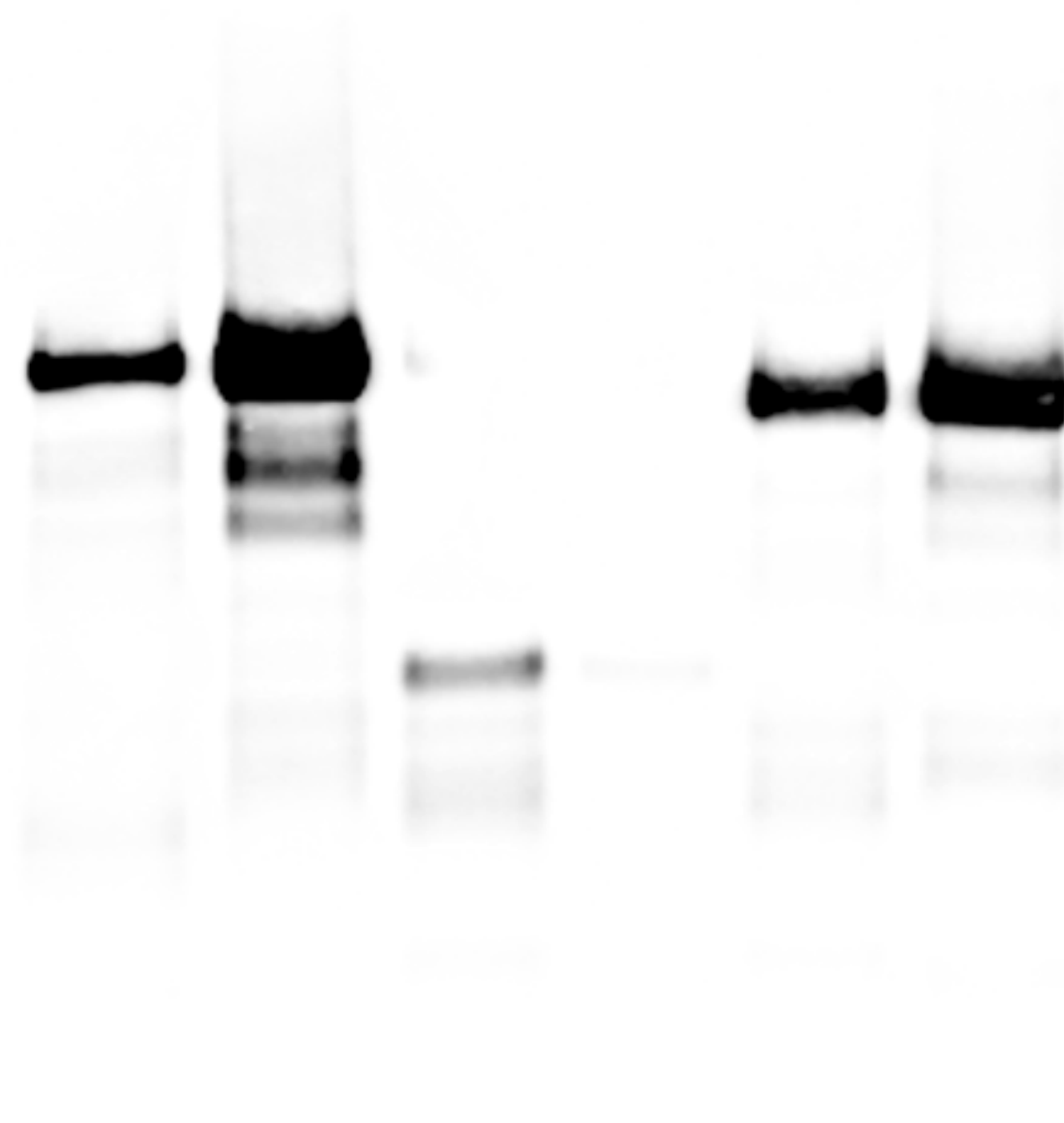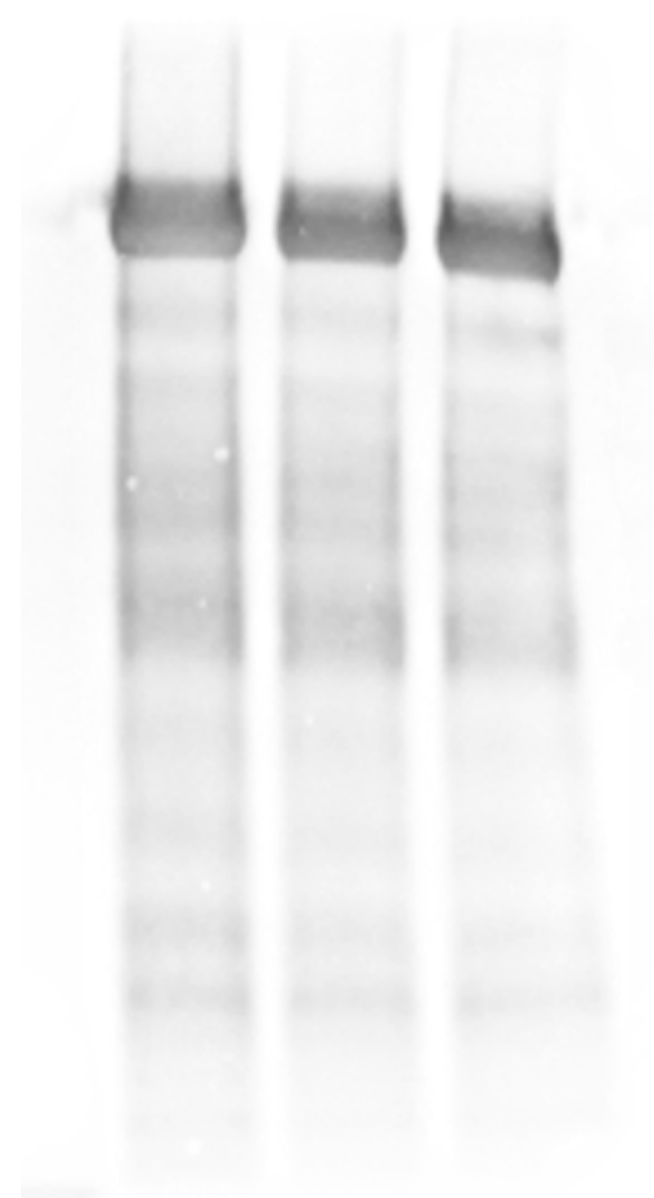

C

Anti-FLAG

Anti-HA on IPs

185  
115  
80  
65  
50  
30  
25  
15  
10

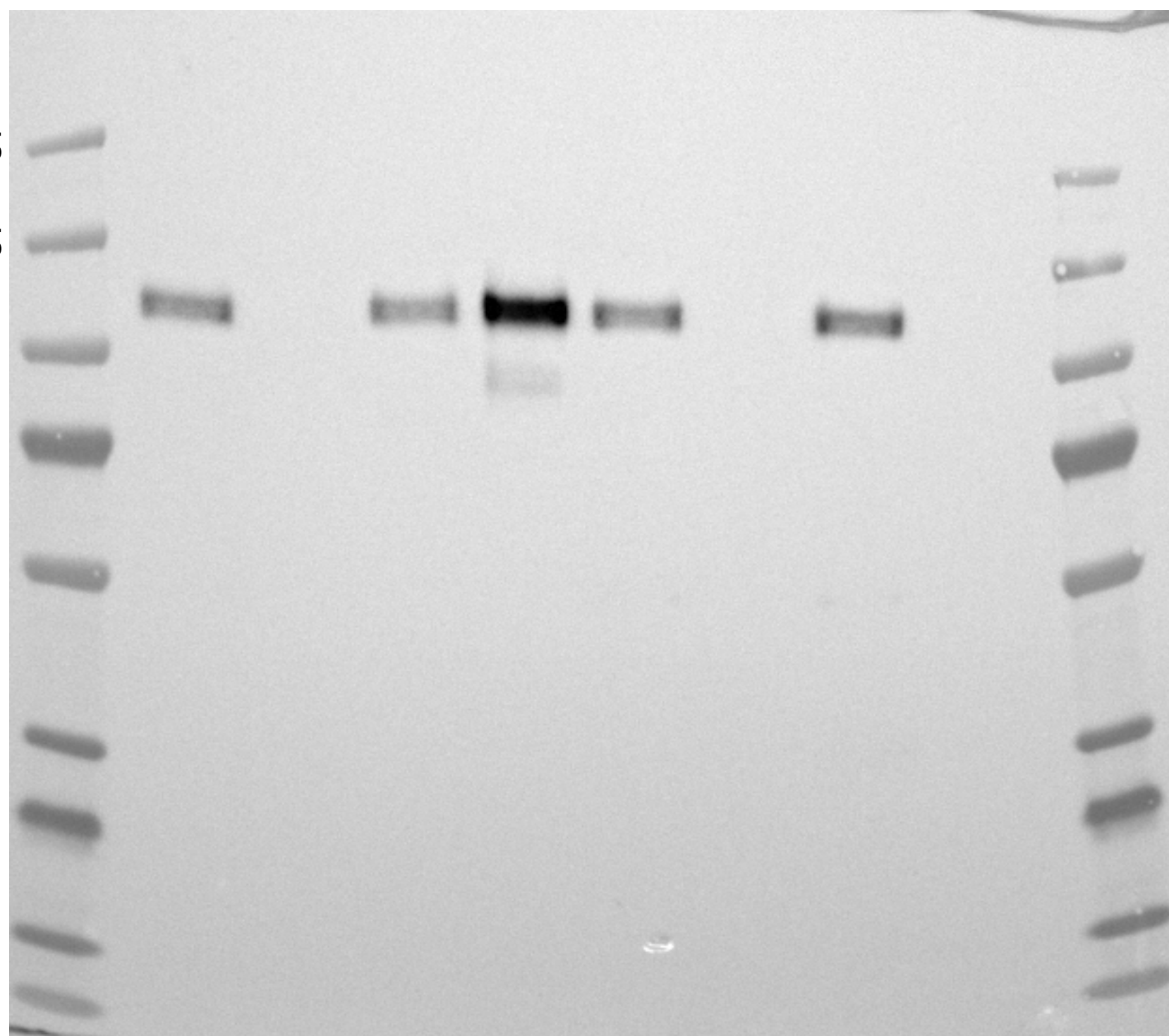

185  
115  
80  
65  
50  
30  
25  
15  
10

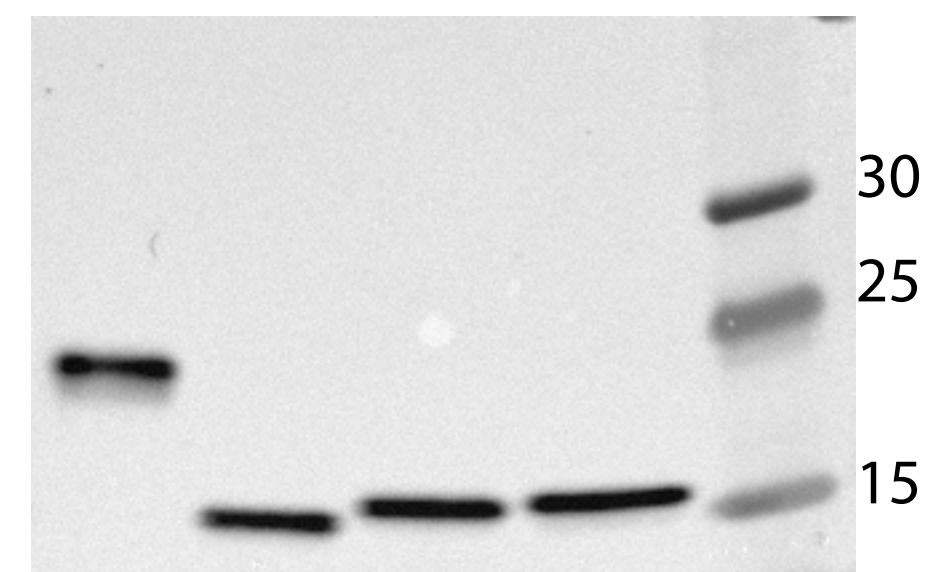

Supplement: Supplementary file 7 — Source Data for Expanded View [file EMBJ-38-e101220-s009.zip › Source_Data_Fig_EV5.pdf]

**B**

**GFP on IPs**

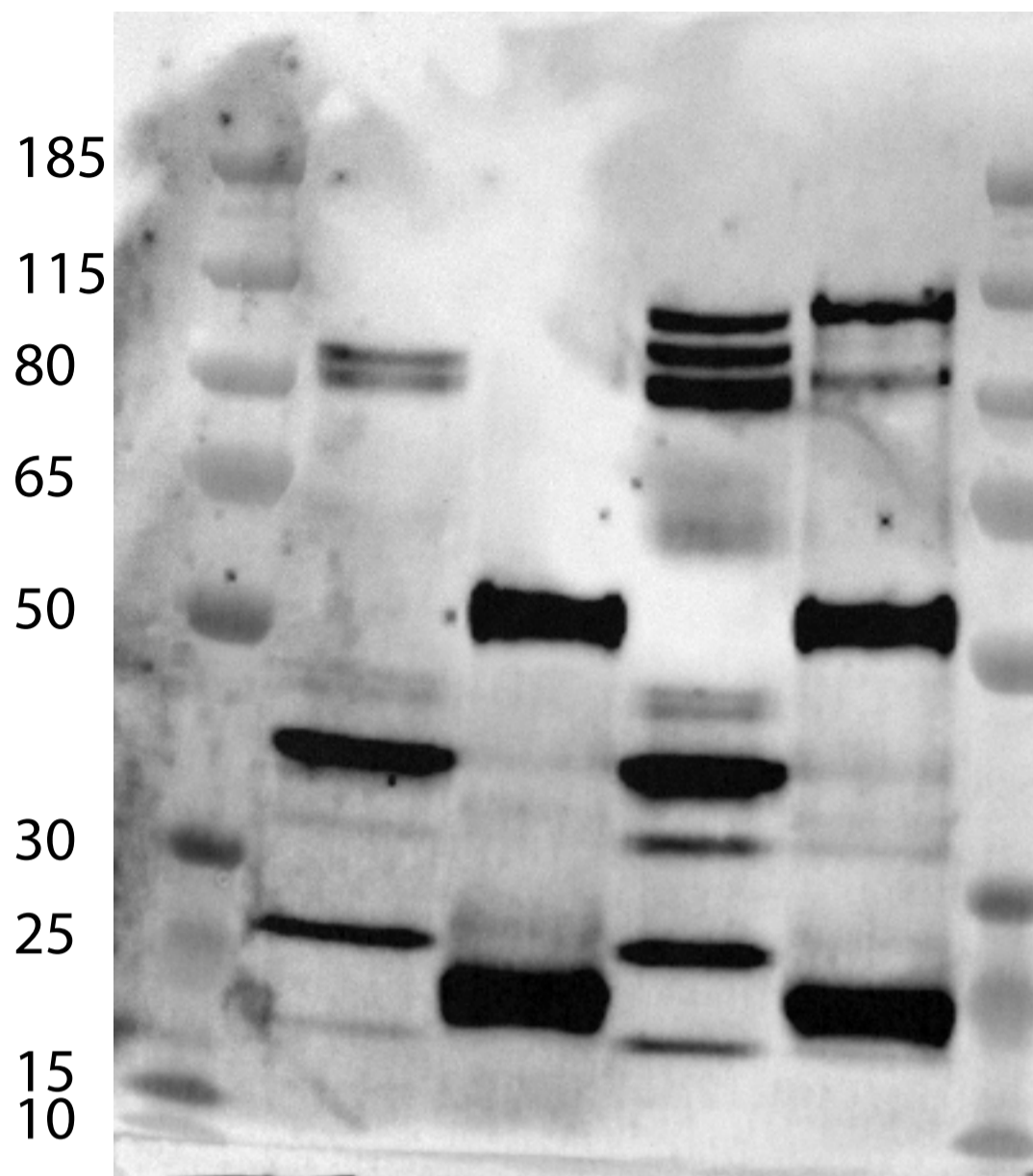

**HA on IPs**

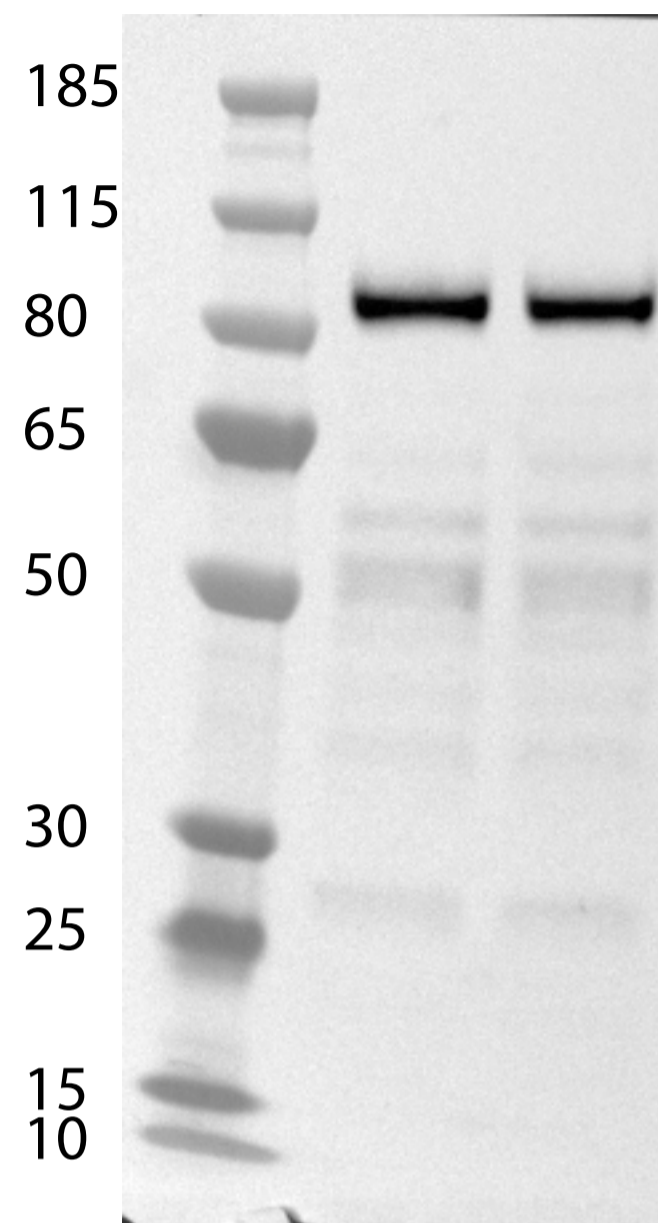

**C**

**FLAG on IPs**

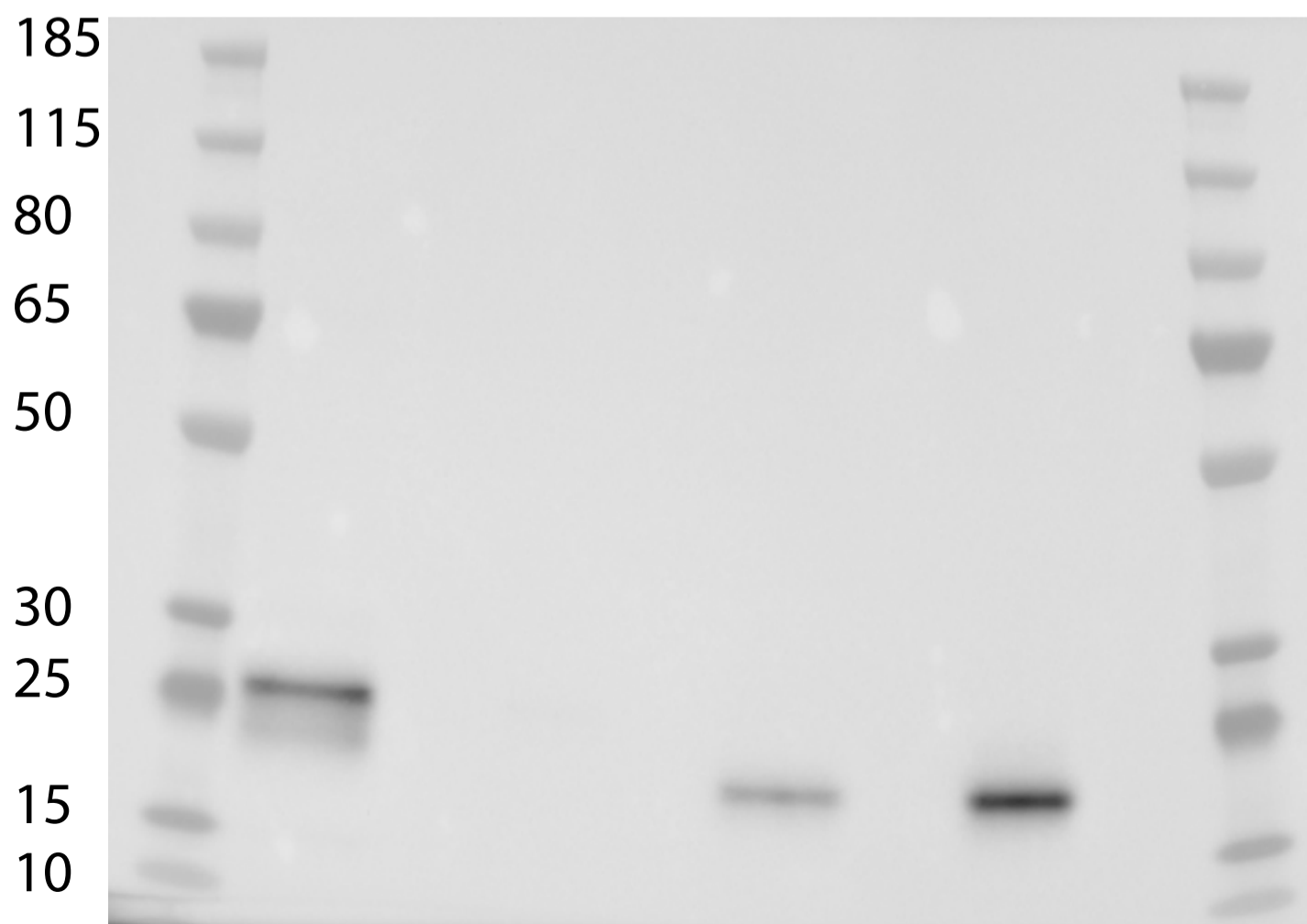

**HA on IPs**

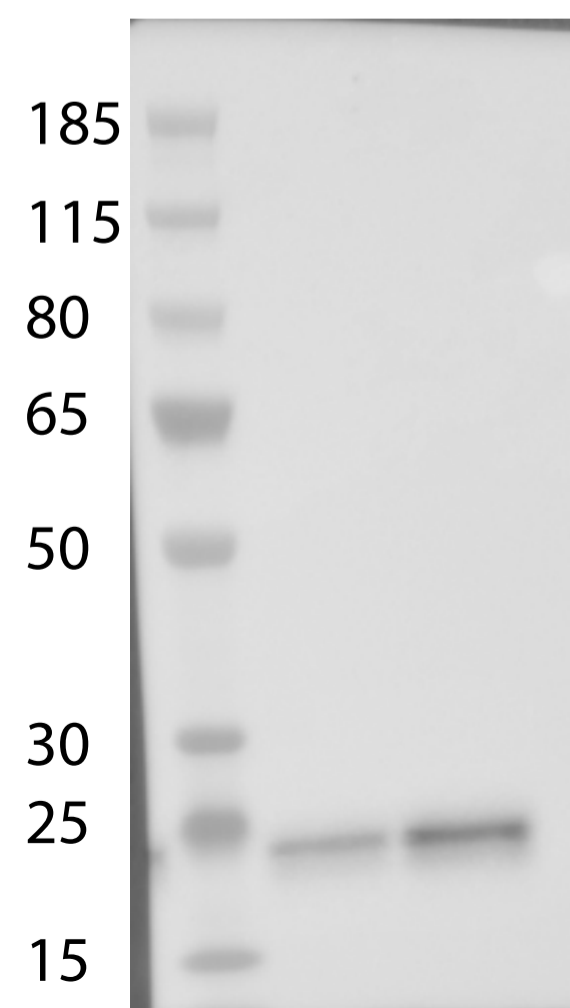

Supplement: Supplementary file 9 — Source Data for Figure 2 [file EMBJ-38-e101220-s007.pdf]

B

Coelacanth KAP1 - FLAG on IPs

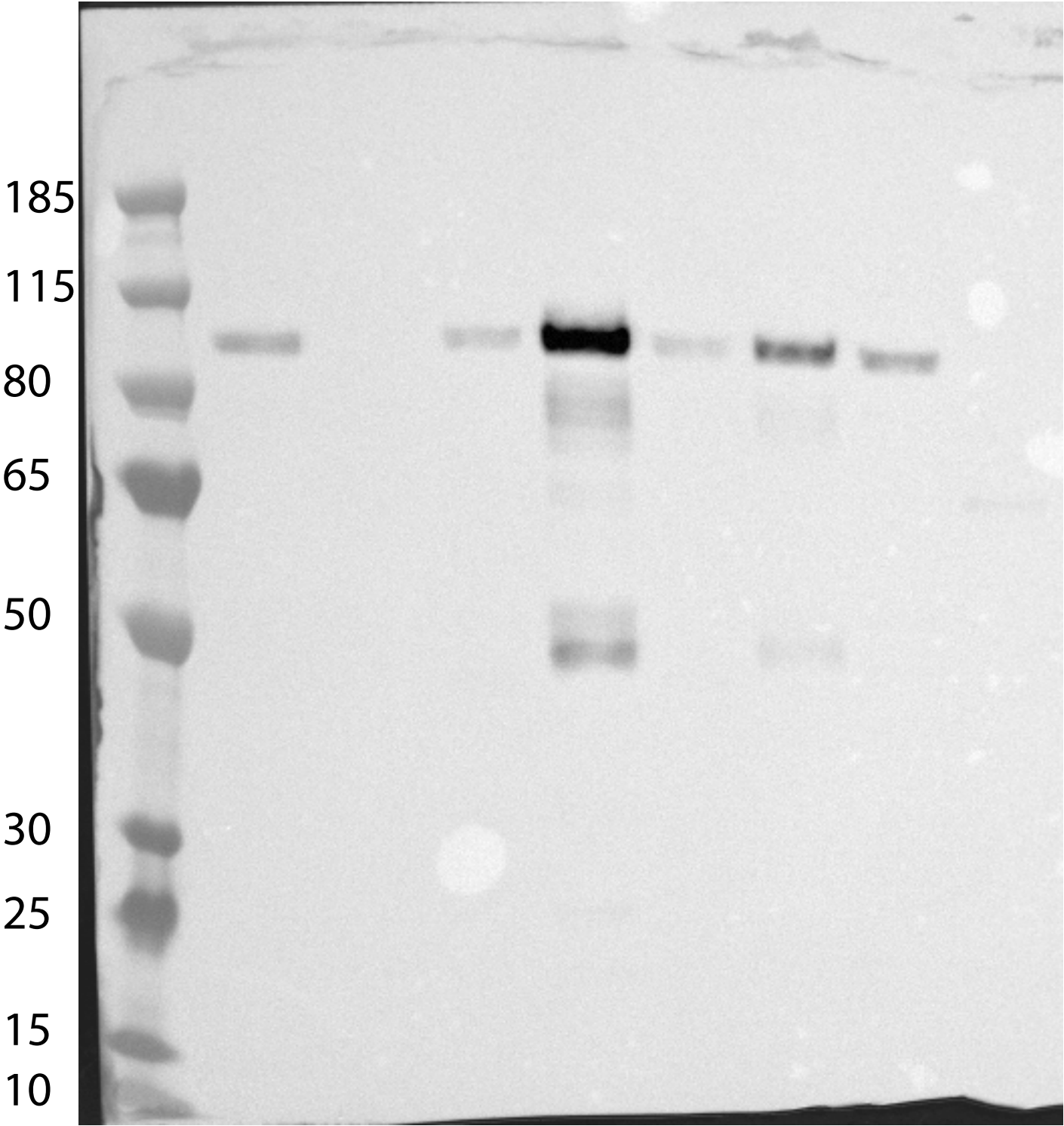

Human KAP1 - FLAG on IPs

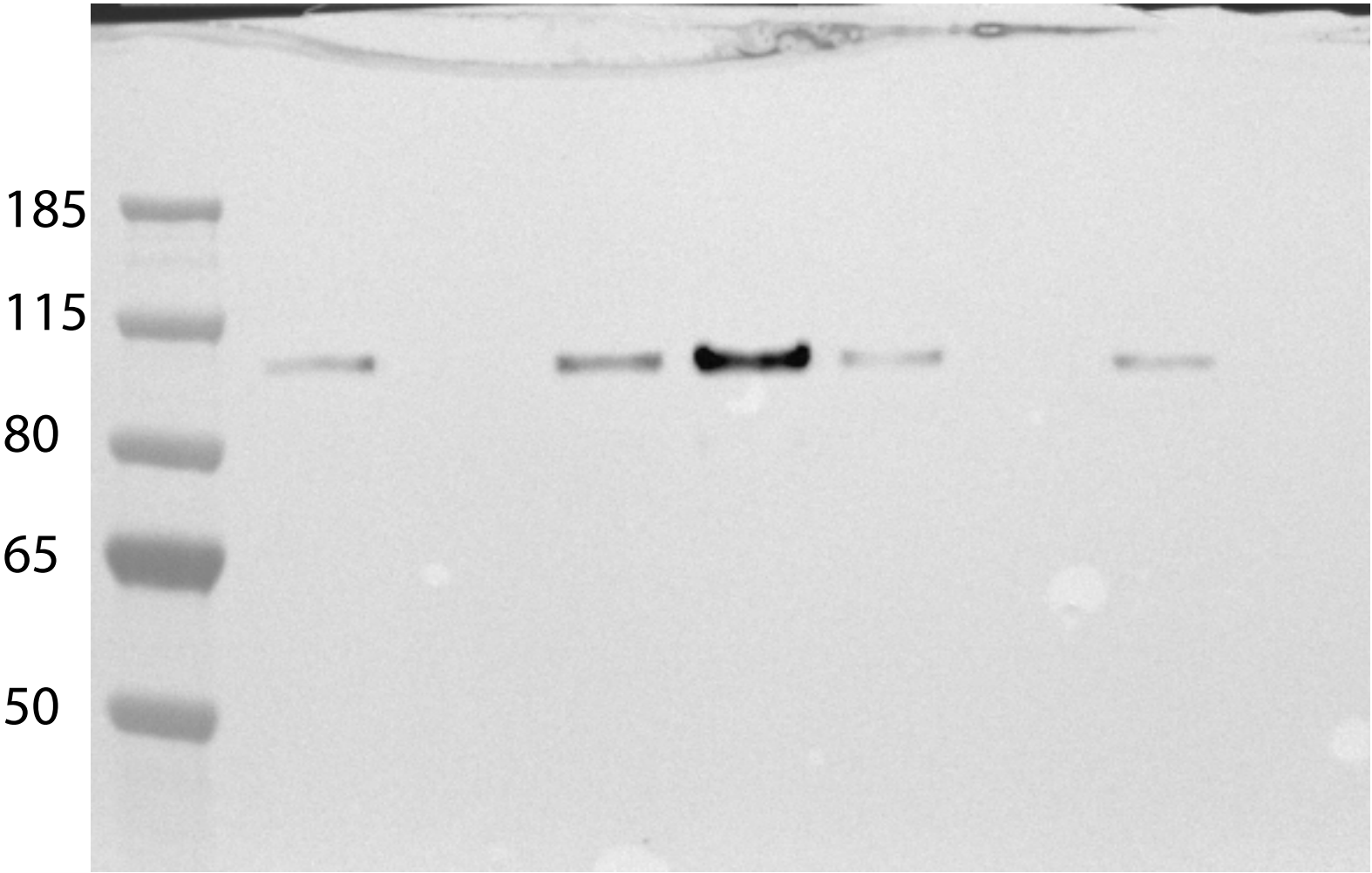

HA on IPs

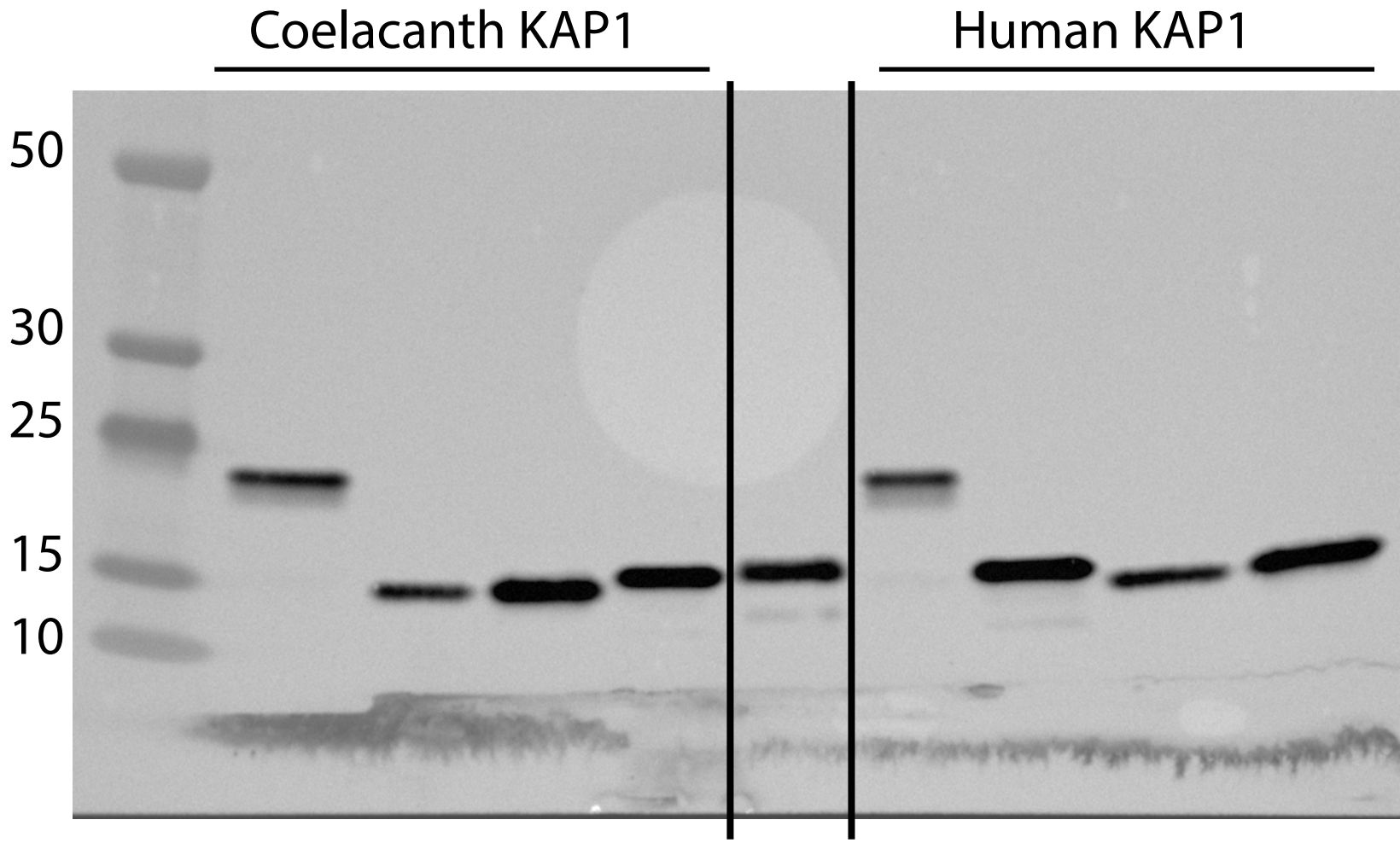

Supplement: Supplementary file 10 — Source Data for Figure 6 [file EMBJ-38-e101220-s008.pdf]
